# Mutations Defining Patient Cohorts With Elevated PD-L1 Expression in Gastric Cancer

**DOI:** 10.3389/fphar.2018.01522

**Published:** 2019-01-08

**Authors:** Otília Menyhárt, Lőrinc Sándor Pongor, Balázs Győrffy

**Affiliations:** ^1^2nd Department of Pediatrics, Semmelweis University, Budapest, Hungary; ^2^MTA TTK Lendület Cancer Biomarker Research Group, Institute of Enzymology, Hungarian Academy of Sciences, Budapest, Hungary

**Keywords:** immunotherapy, stomach cancer, immune checkpoint inhibitors, CD274, PIK3CA, TTK, KIF15

## Abstract

The immunotherapy agent pembrolizumab has been approved for gastric cancer (GC) patients with recurrent or advanced disease who are PD-L1 positive. Mutations in the primary lesion may drive the expression of immune targets thereby priming the tumor to therapeutic sensitivity. In this study, we aimed to uncover mutations associated with elevated PD-L1 expression in GC patients. Data from 410 GC patients were available, including the mutational spectrum of 39,916 genes and expression values of 20,500 genes. PD-L1 gene expression was compared to the mutational status of each gene separately by using a Mann-Whitney *U*-test and a Receiver Operating Characteristic test. Only mutations with a prevalence over 5% were considered. Significance was accepted in cases of *p* < 1E-05 and a fold change over 1.44. Mutations in 209 genes were associated with increased PD-L1 expression. These mutations were enriched in genes related to microtubule-based movement (*p* = 3.4E-4), cell adhesion (*p* = 4.9E-4), response to DNA-damage (*p* = 6.9E-4), and double-strand break-repair (*p* = 1.6E-3). Mutations in *TTK* (*p* = 8.8E-10, AUC = 0.77), *COL7A1* (*p* = 2.0E-9, AUC = 0.74), *KIF15* (*p* = 2.5E-9, AUC = 0.75), and *BDP1* (*p* = 3.3E-9, AUC = 0.74) had the strongest link to elevated PD-L1 expression. Finally, we established a decision tree based on mutations in *PIK3CA, MEF2C, SLC11A1*, and *KIF15* capable to separate patient sub-cohorts with elevated PD-L1 expression. In summary, we identified mutations associated with elevated PD-L1 expression that facilitate the development of better prognostic biomarkers for GC, and might offer insight into the underlying tumor biology.

## Introduction

Gastric cancer (GC) is the fifth most common cancer and the third leading cause of cancer-related mortality in both sexes worldwide with the highest mortality being observed in Eastern Asia, Central and Eastern Europe (Ferlay et al., 2015). Moreover, despite a steady decline in gastric cancer related mortality in the Western hemisphere (Malvezzi et al., 2010), population aging, a distinctive feature of developed countries, contributes once again to increasing trends (Menyhart et al., 2018). Early diagnosis is difficult due to lack of symptoms, particularly in countries without active screening programs, while detection in an advanced stage limits survival prospects (Seeruttun et al., 2017). For advanced patients, standard treatment options based on combined chemotherapy regimens provide limited benefits, and the median overall survival is <12 months (Cunningham et al., 2010). In recent years, immune checkpoint inhibitors (ICI) have rapidly gained momentum in the treatment of advanced GCs and gastroesophageal junction cancers (GEJC) (Taieb et al., 2018).

The immune checkpoint receptor programmed cell death-1 (PD-1) is expressed on activated T cells and prevents overstimulation of immune responses (Francisco et al., 2010), while its ligand, PD-L1, is expressed on tumor infiltrating immune cells and tumor cells. The PD-1/PD-L1 pathway plays an active role in tumor immune evasion (Henick et al., 2014). Blocking their interaction resurrects T-cell-mediated anti-tumor immunity, providing a survival benefit in various advanced, refractory malignancies (Alsaab et al., 2017). The FDA granted accelerated approval to the anti-PD-1 monoclonal antibody pembrolizumab in 2017 as third line treatment for patients with recurrent, locally advanced or metastatic PD-L1-positive GC/GEJC (Fuchs et al., 2018). The anti-PD-1 agent nivolumab demonstrated survival benefits in refractory unresectable advanced or recurrent GC/GEJC, irrespective of PD-L1 expression status, leading to regulatory approval in Japan (Kang et al., 2017).

PD-1 and PD-L1 are expressed in up to 50% of GC/GEJC tumors and are usually associated with the poorest prognosis (Wu et al., 2015). PD-L1 expression is a potential predictive biomarker for the effectiveness of anti-PD-1 therapy: the objective response rate (ORR) to pembrolizumab monotherapy was 16% in PD-L1-positive vs. 6% in PD-L1-negative GC/GEJC patients. Responses were remarkably better when ICIs were administered as a first-line treatment: the ORR reached 36% in PD-L1-positive patients treated with pembrolizumab monotherapy (Fuchs et al., 2018).

PD-L1 status is typically detected by immunohistochemistry. Scoring methods, antibodies and cut-off values are different across clinical studies, making comparison difficult (Teng et al., 2018). Thus, additional biomarkers capable of identifying a subset of patients with elevated PD-L1 (*CD274*) expression as potential candidates for anti-PD-1 therapy are highly in demand.

Genetic alterations within tumors may influence immune system engagement eventually also impacting therapy response; in non-small cell lung cancer (NSCLC) cell lines *EGFR* mutations or EML4-ALK fusions activate the PD-1/PD-L1 pathway via PD-L1 upregulation, inducing immune escape (Akbay et al., 2013; Ota et al., 2015). Accordingly, anti-PD-L1 therapy induced higher ORRs in PD-L1-positive *EGFR* mutant patients (31%) compared to *EGFR* wild-type (22%) NSCLC patients (Peters et al., 2017). *KRAS* mutant advanced NSCLC patients with simultaneous *KEAP1*/*NFE2L2* mutations have reduced PD-L1 expression levels (Skoulidis et al., 2015), which eventually lead to decreased overall survival after the initiation of immune therapy (Arbour et al., 2018). In this study, our aim was to identify genetic alterations in GC that are associated with PD-L1 upregulation. These genes might serve as positive biomarkers capable of identifying responsive tumors. We also combined multiple genes with the goal of creating a decision tree to assist the selection of potentially eligible candidates for early anti-PD-1 therapy.

## Methods

### Sequencing and Expression Database

Mutation and expression data were obtained from the TCGA repository (https://portal.gdc.cancer.gov/). Mutations identified with the mutect2 algorithm were downloaded in VCF format. Variants were selected based on the mutect2 “PASS” status and filtered for mutations with at least 50× overall coverage and a minimum of 5 reads supporting the alteration. The remaining mutations were annotated using the *snpEff* (Cingolani et al., 2012) program using the GRCh38 human genome version. Only the canonical isoforms were selected in the database construction. The expression database was normalized using the DESeq2 (Varet et al., 2016) algorithm.

### Classification Algorithm

Gene expression for PD-L1 was compared to the mutational status of each gene separately using a non-parametric Mann-Whitney U-test and a Receiver Operating Characteristic analysis. Only mutations with a prevalence over 5% were considered. Because of the high number of genes evaluated, statistical significance was only accepted in case of *p* < 1e-05 and a fold change (FC) difference over 1.44. In addition, sensitivity, specificity, and area under the curve (AUC) values were computed for each gene.

Gene ontology analysis for the frequently mutated genes was performed using the Database for Annotation, Visualization and Integrated Discovery (DAVID) Bioinformatics Resource 6.8 to determine the biological meanings of functionally related gene groups (Huang Da et al., 2009). Step-up multiple testing correction was executed for multiple hypothesis testing (Gyorffy et al., 2005).

### Decision Tree

A decision tree was calculated using the conditional inference tree method (Hothorn et al., 2006; Hothorn and Zeileis, 2015). The algorithm uses statistics measuring the association between responses and covariates. In the analysis, we used the univariate distribution to determine the significance. We set the maximum depth to 3 for the tree, and at least 5% of the samples were needed to establish a terminal node during the tree generation. The displayed tree includes the branched decision pipeline and the expression range of PD-L1 in the designated patient cohorts.

## Results

### Database Setup

Data from 438 patients diagnosed with gastric cancer were available from the TCGA repository (https://cancergenome.nih.gov/). Most patients were diagnosed in clinical stage III and with grade 3 disease. 64% of the patients were male and 69% of patients were 60 years of age or older, with a median age of 67 years. The average follow-up time was 9.86 months, and 20% of patients died during this period. Over 8% of the patients were identified with residual disease, while pathological complete response (pCR) following adjuvant therapy occurred in 32.4% of the patients (for details see Supplemental Table 1).

### Mutations Associated With PD-L1 Expression

On average, 873 mutation events were identified per patient in our population based on the mutational profile of 39,916 genes. The most frequently mutated genes, *PCDHA1-PCDHA4* and the tumor suppressor *TP53*, were mutated on average in every second patient.

**Figure 1:**
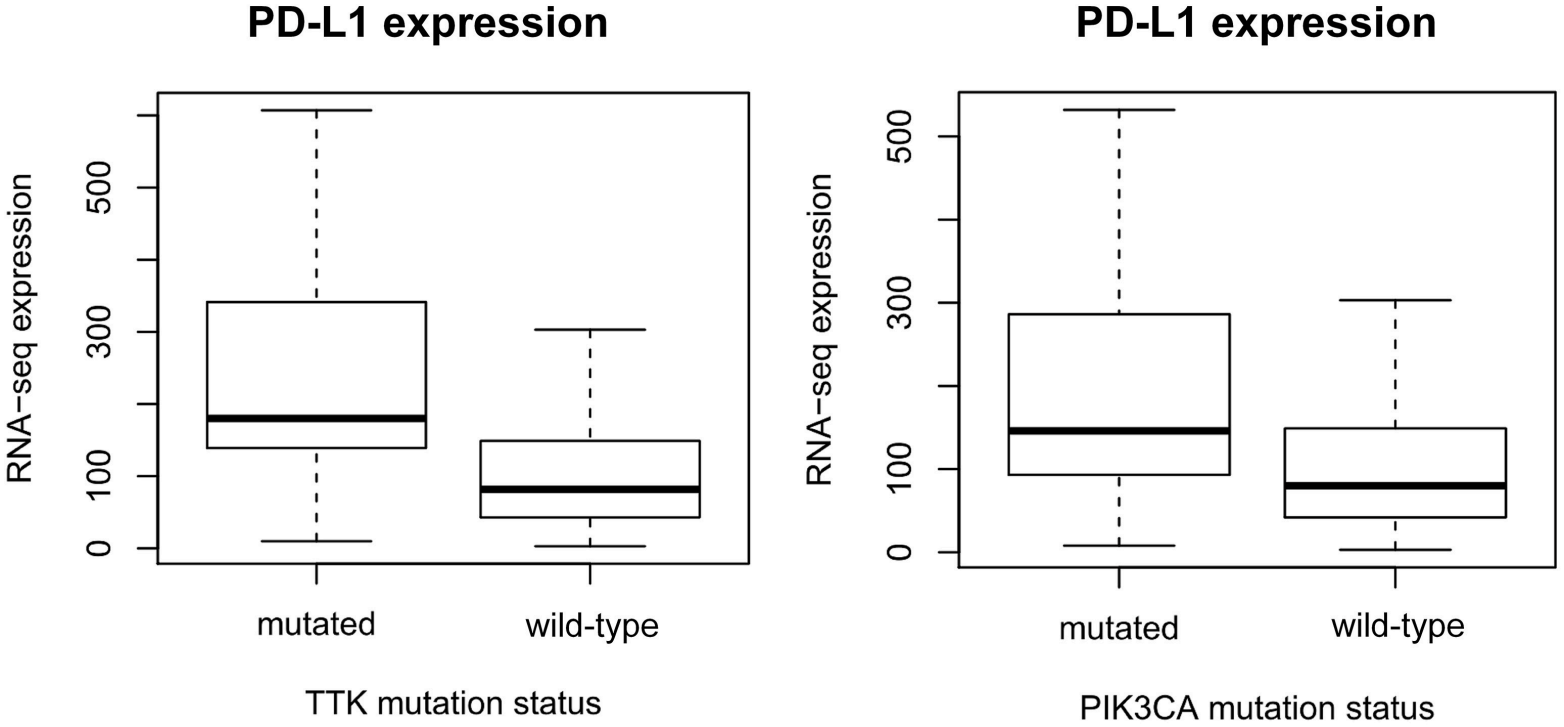
Gene mutations defining higher PD-L1 expression. mRNA levels of PD-L1 (*CD274*) are significantly higher in *TTK* (*p* = 8.8E-10) and *PIK3CA* (*p* = 1.7E-08) mutant patients. The plots show Q1/Q2/Q3 within min–max range.

The expression levels of 20,500 genes were investigated in our patient population. Data consisting of both the mutational spectrum and expression values for all genes were available for 410 GC patients. Mutations in 209 genes were associated with significantly increased PD-L1 expression (Supplemental Table 2). Mutations in *TTK* (*p* = 8.83E-10, AUC = 0.77), *COL7A1* (*p* = 2E-9, AUC = 0.74), *KIF15* (*p* = 2.49E-9, AUC = 0.75), and *BDP1* (*p* = 3.26E-9, AUC = 0.75) presented the strongest link to elevated PD-L1 expression (Figure 1).

We performed gene enrichment analysis to determine the biological functions of the most frequent mutations. According to the GO analysis, the significantly mutated genes were involved in microtubule-based movement (*p* = 3.4E-4), cell adhesion (*p* = 4.9E-4), response to DNA-damage (*p* = 6.9E-4), regulation of gene expression (*p* = 1.5E-4), and homologous recombination-dependent double-strand break repair (*p* = 1.6E-3) (Supplemental Table 3).

### Mutation-Based Hierarchical Clustering

**Figure 2:**
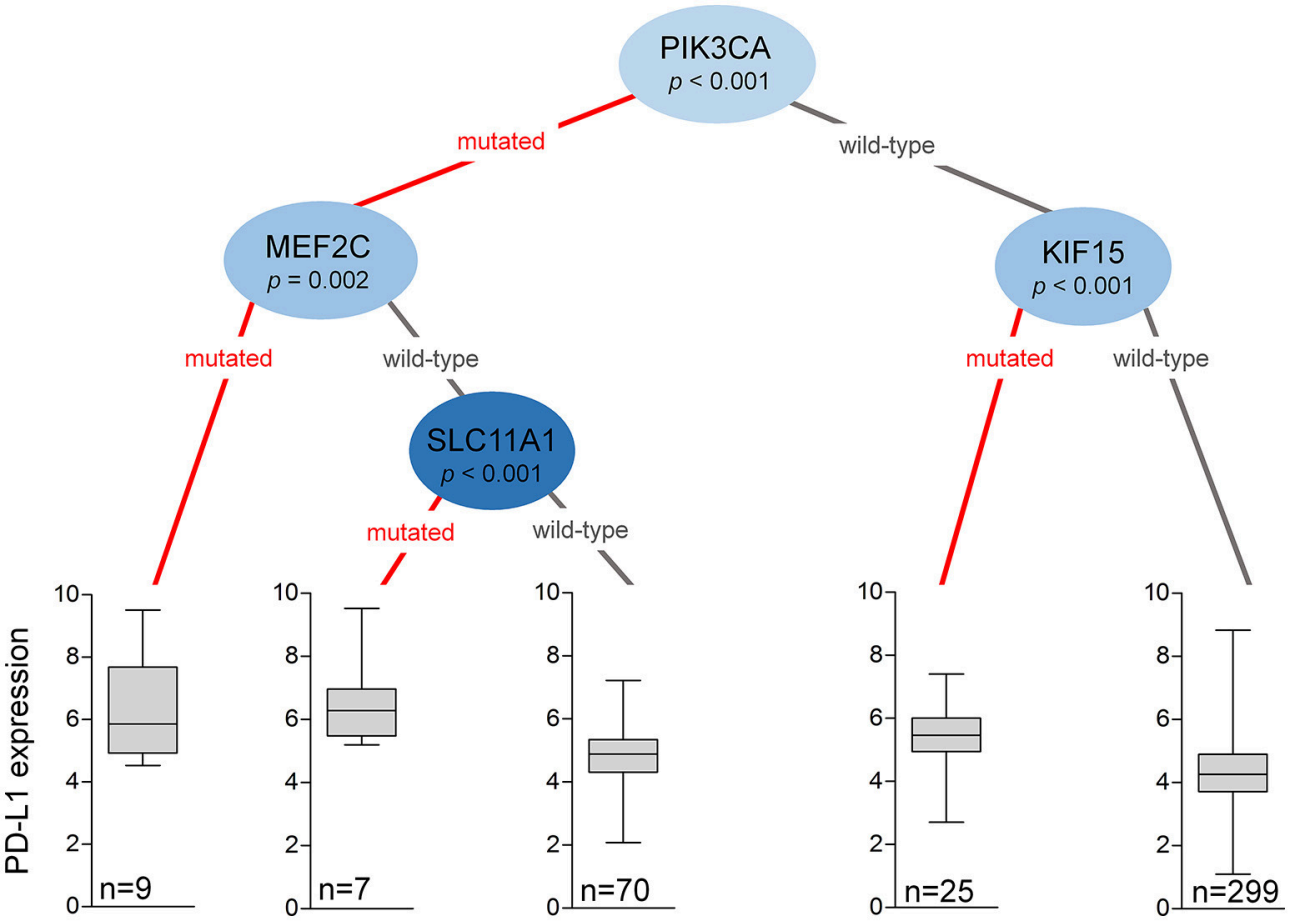
Mutations in the *PIK3CA, MEF2C, SLC11A1*, and *KIF15* genes help to stratify patients into subcohorts with dissimilar PD-L1 (*CD274*) expression. The decision tree was generated by analyzing the mutational status of all genes simultaneously with a minimal threshold of having at least 5% of the patients in each node. The plots show Q1/Q2/Q3 within min–max range.

The mutational status of multiple critical genes may assist in the selection of even stronger candidates for ICI therapy. Based on hierarchical clustering of all significant genes with mutational prevalence >5% (when considering the mutation as a terminal node) and FC of at least 1.44, we constructed a decision tree to stratify patients with differential PD-L1 expression (Figure 2). The mutational status of *PIK3CA* was the best performing root node dividing patients into major subclasses. Both *PIK3CA* wild-type and mutant populations could be subdivided using additional mutations. Approximately 73% of all patients harbored wild-type alleles of both *PIK3CA* and *KIF15* that are associated with significantly lower overall PD-L1 expression, while *PIK3CA* wild-type patients with *KIF15* mutations (6%) showed significantly elevated PD-L1 expression (*p* < 1e-03). Patients with *PIK3CA* mutations (21%) could be stratified by two further genes. The presence of *MEF2C* (*p* = 0.002) or *SLC11A1* (*p* < 0.001) mutations (4%) was linked to PD-L1 upregulation, while PD-L1 expression was lower in subjects with the wild-type alleles of *SLC11A1* (17%). Altogether 10% of all patients harbored mutations associated with PD-L1 overexpression.

## Discussion

Genetic aberrations within tumors may alter PD-1/PD-L1 interactions by modulating the expression of immune markers (Skoulidis et al., 2015) potentially affecting therapy response (Arbour et al., 2018). We identified mutations of 209 genes associated with PD-L1 upregulation in GC that are involved in functions, such as microtubule-based movement, cell adhesion, gene expression regulation, response to DNA damage and double-strand break repair. Mutations in the *TTK, COL7A1, KIF15*, and *BDP1* genes present the strongest association with elevated PD-L1 expression. *TTK* frameshift mutations appear in microsatellite instability-high (MSI-H) subtypes of GC that may alter cell cycle regulation (Ahn et al., 2009). Nonetheless, understanding the exact role of these genes in PD-L1 regulation requires further investigations.

To promote patient stratification, we created a decision tree capable of hypothetically prioritizing candidates for ICI therapy. The root node is set up by *PIK3CA*, while mutations involving *MEF2C, SLC11A1*, and *KIF15* provide additional sorting, all known to modulate various aspects of the immune system. *MEF2C* plays a role in immunity and leukemia development (Schuler et al., 2008), and was implicated as an oncogene in various hematological and solid cancers (Pon and Marra, 2016). *SLC11A1* encodes a transmembrane proton/divalent cation symporter, and participates in innate defense against pathogens by influencing macrophage activation (Archer et al., 2015). *KIF15* is involved in the maintenance of the mitotic spindle, and is upregulated in multiple solid malignancies (Scanlan et al., 2001; Wang et al., 2017). *KIF15* also inhibits the endocytic trafficking of α2 integrin, implicated in various immune diseases (De Fougerolles et al., 2000). Except for *PIK3CA*, the functional relationship between the described mutations, PD-L1 upregulation and GC outcome is yet unexplored.

Our findings are in keeping with previous reports showing that gastric tumors with high PD-L1 expression levels frequently harbor *PIK3CA* mutations (Cancer Genome Atlas Research Network, 2014). In fact, *PIK3CA* is among the most frequently mutated genes in GC, present in ~32% of hypermutated and 12% of non-hypermutated tumors (Cancer Genome Atlas Research Network, 2014; Cristescu et al., 2015). *PIK3CA* mutations are associated with more aggressive features, such as advanced T stage, poor differentiation and vascular invasion, especially in locoregional disease (Kim et al., 2017), and higher CD8^+^ T cell infiltration (Siemers et al., 2017). At the same time, *PIK3CA* mutations have not been directly linked to patient prognosis (Harada et al., 2016; Kim et al., 2017). In this study, we found diversity within the *PIK3CA* mutant population, as additional genes were required to stratify patients based on differential PD-L1 expression.

The PI3K/Akt-pathway is involved in the immune response against malignant cells (Dituri et al., 2011), and increases the expression of immune markers. Inhibiting PI3K in melanoma cells reduced (Jiang et al., 2013), and knockdown of PTEN in colorectal cancer cell lines increased the expression of PD-L1 (Song et al., 2013). The PI3K/Akt-pathway regulates PD-L1 expression on a cell- and tissue-dependent manner by either transcriptional or post-transcriptional mechanisms (Song et al., 2013).

*PIK3CA* mutations appear with high frequency in Epstein-Barr virus positive and MSI-high subtypes of GC (Cancer Genome Atlas Research Network, 2014; Cristescu et al., 2015), and *TTK* frameshift mutations are also relatively frequent in the latter (Ahn et al., 2009). These particular GC subtypes have been suggested to be the most promising candidates for immunotherapy (Cancer Genome Atlas Research Network, 2014; Cristescu et al., 2015). In a recent clinical trial, MSI-high patients treated with ICI reached higher ORRs compared to patients with non-MSI-high tumors. However, the prevalence of MSI-high cases reached only 4% in the study population (Fuchs et al., 2018). Future trials will be required to clarify the subgroup specific responses to anti-PD-1 therapy.

In summary, we present an approach to narrow the list of potentially eligible patients for early anti-PD-1 therapy, and provide a foundation for future studies to reveal functional implications of key mutations on PD-L1 regulation. Nevertheless, the observed associations do not infer functional relationships. Our results facilitate the development of prognostic biomarkers for GC, and offer insight into the underlying tumor biology.

## Author Contributions

BG contributed to the conception and design of the study. LP organized the data acquisition and analysis. OM wrote the first draft of the manuscript. LP and BG wrote sections of the manuscript. All authors contributed to manuscript revision, read and approved the submitted version.

### Conflict of Interest Statement

The authors declare that the research was conducted in the absence of any commercial or financial relationships that could be construed as a potential conflict of interest.
