# Supplementary material for: Mutations Defining Patient Cohorts With Elevated PD-L1 Expression in Gastric Cancer
Source: Front Pharmacol. 2019 Jan 8;9:1522. doi: 10.3389/fphar.2018.01522 (PMC6331584; doi:10.3389/fphar.2018.01522)
Supplement: Supplementary file 1 [file Table_1.DOCX]

**Supplemental Table 1. Clinical characterization of GC/GEJC patients from the TCGA dataset.**

| **Characteristics** | | **Participants** | **(%)** |
| --- | --- | --- | --- |
| **Sex** | Male | 281 | 64.2 |
|  | Female | 157 | 35.8 |
| **Age/years** | <60 | 130 | 29.7 |
|  | ≥ 60 | 303 | 69.2 |
|  | Unknown | 5 | 1.1 |
| **Clinical stage** | I | 59 | 13.5 |
|  | II | 131 | 29.9 |
|  | III | 185 | 42.2 |
|  | IV | 45 | 10.3 |
|  | N/A | 18 | 4.1 |
| **Grade** | I | 12 | 2.7 |
|  | II | 159 | 36.3 |
|  | III | 258 | 58.9 |
|  | N/A | 9 | 2.1 |
| **Residual tumor after surgery** | | 37 | 8.4 |
|  | No data | 54 | 12.3 |
| **Tumor site** | Fundus/body | 151 | 34.5 |
|  | Antrum/distal | 159 | 36.3 |
|  | Cardia/proximal | 61 | 13.9 |
|  | Gastroesophageal junction | 46 | 10.5 |
|  | Unknown | 21 | 4.8 |
| **Therapy** | Antireflux | 43 | 9.8 |
|  | Adjuvant radiation | 51 | 11.6 |
|  | Molecular targeted | 101 | 23.1 |
| **Response** | pCR | 142 | 32.4 |
| **Death event** | | 87 | 19.9 |
