# Supplementary material for: Mutations Defining Patient Cohorts With Elevated PD-L1 Expression in Gastric Cancer
Source: Front Pharmacol. 2019 Jan 8;9:1522. doi: 10.3389/fphar.2018.01522 (PMC6331584; doi:10.3389/fphar.2018.01522)
Supplement: Supplementary file 2 [file Table_2.DOCX]

**Supplemental Table 2. List of mutations significantly associated with increased PD-L1 (*CD274*) expression.**

Abbreviations: #_mut – number of mutant patients in the dataset. FC - Fold Change: magnitude of PD-L1 expression difference between mutant and wild-type, AUC - Area Under the Curve

| **Gene symbol** | **#_mut** | **#_wild** | **FC** | ***p*-value** | **AUC** | **sensitivity** | **specificity** |  | SLK | 31 | 379 | 2.51 | 2.70E-06 | 0.75 | 0.69 | 0.74 |
| --- | --- | --- | --- | --- | --- | --- | --- | --- | --- | --- | --- | --- | --- | --- | --- | --- |
| TTK | 51 | 359 | 2.2 | 8.80E-10 | 0.77 | 0.73 | 0.76 |  | PTPN23 | 35 | 375 | 1.87 | 2.70E-06 | 0.74 | 0.63 | 0.77 |
| COL7A1 | 61 | 349 | 2.1 | 2.00E-09 | 0.74 | 0.67 | 0.79 |  | KCNT1 | 48 | 362 | 1.86 | 2.80E-06 | 0.71 | 0.73 | 0.58 |
| KIF15 | 55 | 355 | 2.2 | 2.50E-09 | 0.75 | 0.63 | 0.82 |  | EFNB3 | 23 | 387 | 2.51 | 2.80E-06 | 0.79 | 0.71 | 0.83 |
| BDP1 | 53 | 357 | 2.18 | 3.30E-09 | 0.75 | 0.73 | 0.70 |  | C3 | 52 | 358 | 1.85 | 2.90E-06 | 0.70 | 0.51 | 0.85 |
| KIAA1109 | 86 | 324 | 2.13 | 3.70E-09 | 0.71 | 0.76 | 0.63 |  | COL4A6 | 45 | 365 | 1.9 | 2.90E-06 | 0.71 | 0.59 | 0.78 |
| ULK4 | 57 | 353 | 2.11 | 6.20E-09 | 0.74 | 0.73 | 0.67 |  | RPS6KB2 | 28 | 382 | 1.91 | 2.90E-06 | 0.76 | 0.64 | 0.89 |
| ACACA | 65 | 345 | 2.15 | 6.80E-09 | 0.73 | 0.74 | 0.68 |  | CPT1C | 34 | 376 | 2.08 | 2.90E-06 | 0.74 | 0.74 | 0.71 |
| DNAH2 | 61 | 349 | 2.2 | 1.40E-08 | 0.73 | 0.74 | 0.66 |  | STRADA | 27 | 383 | 2.19 | 3.00E-06 | 0.77 | 0.71 | 0.78 |
| PCSK5 | 55 | 355 | 2.07 | 1.60E-08 | 0.74 | 0.61 | 0.80 |  | ZW10 | 38 | 372 | 2.07 | 3.00E-06 | 0.73 | 0.72 | 0.76 |
| PIK3CA | 86 | 324 | 1.82 | 1.70E-08 | 0.70 | 0.63 | 0.70 |  | DOCK8 | 39 | 371 | 2.11 | 3.20E-06 | 0.73 | 0.72 | 0.69 |
| FOCAD | 34 | 376 | 2.88 | 2.10E-08 | 0.79 | 0.73 | 0.76 |  | RNF43 | 53 | 357 | 1.98 | 3.30E-06 | 0.70 | 0.73 | 0.60 |
| ACVR2A | 72 | 338 | 2.03 | 2.30E-08 | 0.71 | 0.74 | 0.63 |  | SGPL1 | 29 | 381 | 2.64 | 3.40E-06 | 0.76 | 0.69 | 0.72 |
| MCPH1 | 45 | 365 | 2.18 | 2.60E-08 | 0.75 | 0.73 | 0.71 |  | UBA6 | 47 | 363 | 1.98 | 3.50E-06 | 0.71 | 0.71 | 0.70 |
| KIAA0895L | 30 | 380 | 3.16 | 2.70E-08 | 0.80 | 0.75 | 0.83 |  | SRPK2 | 33 | 377 | 1.97 | 3.50E-06 | 0.74 | 0.72 | 0.70 |
| NCAPD3 | 51 | 359 | 2.18 | 3.00E-08 | 0.74 | 0.74 | 0.69 |  | EVI5 | 28 | 382 | 2.99 | 3.50E-06 | 0.76 | 0.71 | 0.82 |
| SCAF4 | 46 | 364 | 2.43 | 3.40E-08 | 0.75 | 0.81 | 0.63 |  | KIF9 | 30 | 380 | 2.11 | 3.70E-06 | 0.75 | 0.71 | 0.77 |
| KMT2D | 74 | 336 | 2.12 | 3.50E-08 | 0.70 | 0.74 | 0.62 |  | NR2C1 | 24 | 386 | 2.12 | 3.80E-06 | 0.78 | 0.71 | 0.88 |
| PDE8A | 45 | 365 | 2.21 | 4.00E-08 | 0.75 | 0.72 | 0.69 |  | ASPH | 44 | 366 | 2.04 | 3.80E-06 | 0.71 | 0.70 | 0.66 |
| C11ORF65 | 48 | 362 | 2.15 | 5.10E-08 | 0.74 | 0.75 | 0.73 |  | NUP160 | 35 | 375 | 1.97 | 3.80E-06 | 0.74 | 0.71 | 0.71 |
| ZNF462 | 56 | 354 | 2.07 | 5.10E-08 | 0.73 | 0.73 | 0.68 |  | PCDHGA6 | 143 | 267 | 1.76 | 3.90E-06 | 0.64 | 0.66 | 0.59 |
| ORC4 | 69 | 341 | 2.04 | 5.60E-08 | 0.71 | 0.74 | 0.62 |  | PCDHGA7 | 133 | 277 | 1.85 | 3.90E-06 | 0.64 | 0.77 | 0.51 |
| PBRM1 | 43 | 367 | 2.12 | 5.90E-08 | 0.75 | 0.62 | 0.84 |  | PCDHGB4 | 133 | 277 | 1.85 | 3.90E-06 | 0.64 | 0.77 | 0.51 |
| FLNC | 64 | 346 | 2.02 | 6.90E-08 | 0.71 | 0.74 | 0.64 |  | SART3 | 38 | 372 | 1.89 | 3.90E-06 | 0.73 | 0.49 | 0.87 |
| HERC1 | 58 | 352 | 2.12 | 7.70E-08 | 0.72 | 0.74 | 0.67 |  | DNAH8 | 90 | 320 | 1.85 | 4.00E-06 | 0.66 | 0.63 | 0.64 |
| ESRP1 | 44 | 366 | 2.06 | 8.00E-08 | 0.75 | 0.72 | 0.70 |  | ZMYM6 | 37 | 373 | 2.05 | 4.00E-06 | 0.73 | 0.75 | 0.65 |
| TLN2 | 52 | 358 | 2.07 | 8.50E-08 | 0.73 | 0.65 | 0.75 |  | ZZEF1 | 43 | 367 | 2.03 | 4.00E-06 | 0.71 | 0.72 | 0.65 |
| KMT2E | 43 | 367 | 2.21 | 9.40E-08 | 0.75 | 0.77 | 0.65 |  | LMAN1 | 37 | 373 | 2.45 | 4.00E-06 | 0.73 | 0.75 | 0.68 |
| MORC1 | 65 | 345 | 2.09 | 9.50E-08 | 0.71 | 0.73 | 0.62 |  | ABCA4 | 45 | 365 | 2.01 | 4.00E-06 | 0.71 | 0.73 | 0.62 |
| VPS13A | 60 | 350 | 2.1 | 9.80E-08 | 0.72 | 0.73 | 0.67 |  | PLEKHG4 | 29 | 381 | 1.97 | 4.10E-06 | 0.76 | 0.70 | 0.79 |
| TRRAP | 89 | 321 | 1.93 | 1.10E-07 | 0.68 | 0.64 | 0.69 |  | NEMF | 36 | 374 | 2.48 | 4.20E-06 | 0.73 | 0.79 | 0.67 |
| ATM | 76 | 334 | 1.96 | 1.20E-07 | 0.69 | 0.74 | 0.61 |  | SPECC1 | 41 | 369 | 1.88 | 4.20E-06 | 0.72 | 0.72 | 0.66 |
| RAB3GAP1 | 39 | 371 | 2.15 | 1.30E-07 | 0.76 | 0.73 | 0.77 |  | LRBA | 60 | 350 | 1.85 | 4.20E-06 | 0.69 | 0.72 | 0.58 |
| UBR5 | 73 | 337 | 1.9 | 1.50E-07 | 0.70 | 0.63 | 0.68 |  | ZNF609 | 39 | 371 | 2.01 | 4.20E-06 | 0.72 | 0.78 | 0.62 |
| SMARCA2 | 46 | 364 | 2.29 | 1.80E-07 | 0.74 | 0.74 | 0.72 |  | SMC2 | 35 | 375 | 2.03 | 4.60E-06 | 0.73 | 0.72 | 0.74 |
| LRP1 | 69 | 341 | 2.1 | 2.00E-07 | 0.70 | 0.73 | 0.64 |  | SMC6 | 35 | 375 | 2.19 | 4.60E-06 | 0.73 | 0.72 | 0.77 |
| BTAF1 | 46 | 364 | 2.1 | 2.20E-07 | 0.73 | 0.76 | 0.70 |  | ASS1 | 23 | 387 | 2.29 | 4.60E-06 | 0.78 | 0.59 | 0.87 |
| FANCA | 31 | 379 | 2.34 | 2.30E-07 | 0.78 | 0.72 | 0.77 |  | DENND2A | 30 | 380 | 2.09 | 4.60E-06 | 0.75 | 0.66 | 0.80 |
| NPC1 | 50 | 360 | 2.02 | 2.30E-07 | 0.73 | 0.73 | 0.66 |  | KIF5B | 36 | 374 | 2.16 | 4.60E-06 | 0.73 | 0.73 | 0.72 |
| ZMYM4 | 42 | 368 | 2.17 | 2.70E-07 | 0.74 | 0.73 | 0.79 |  | ARID1B | 44 | 366 | 2.04 | 4.70E-06 | 0.71 | 0.63 | 0.75 |
| MTHFD1 | 30 | 380 | 2.64 | 2.80E-07 | 0.78 | 0.72 | 0.83 |  | UBR4 | 76 | 334 | 1.85 | 4.70E-06 | 0.67 | 0.72 | 0.57 |
| WDFY3 | 68 | 342 | 1.93 | 2.80E-07 | 0.70 | 0.74 | 0.63 |  | OPA1 | 26 | 384 | 2.6 | 4.80E-06 | 0.77 | 0.72 | 0.85 |
| TBCD | 52 | 358 | 2.15 | 2.90E-07 | 0.72 | 0.82 | 0.56 |  | ATP2C1 | 33 | 377 | 2.03 | 4.90E-06 | 0.74 | 0.45 | 0.97 |
| BMPR2 | 38 | 372 | 2.14 | 2.90E-07 | 0.75 | 0.72 | 0.74 |  | KDM5B | 48 | 362 | 2.06 | 4.90E-06 | 0.70 | 0.73 | 0.65 |
| LARS | 29 | 381 | 2.55 | 3.00E-07 | 0.79 | 0.75 | 0.79 |  | NCAN | 35 | 375 | 2.01 | 4.90E-06 | 0.73 | 0.50 | 0.91 |
| RERE | 38 | 372 | 2.15 | 3.20E-07 | 0.75 | 0.72 | 0.74 |  | NOP58 | 26 | 384 | 2.11 | 5.10E-06 | 0.77 | 0.72 | 0.77 |
| FBXO18 | 30 | 380 | 2.59 | 3.40E-07 | 0.78 | 0.72 | 0.80 |  | PPM1G | 24 | 386 | 2.72 | 5.10E-06 | 0.78 | 0.71 | 0.79 |
| MDN1 | 83 | 327 | 1.95 | 3.60E-07 | 0.68 | 0.75 | 0.59 |  | DOPEY1 | 38 | 372 | 2.01 | 5.20E-06 | 0.72 | 0.70 | 0.71 |
| POLQ | 65 | 345 | 2.02 | 3.90E-07 | 0.70 | 0.66 | 0.74 |  | RIF1 | 48 | 362 | 1.97 | 5.20E-06 | 0.70 | 0.73 | 0.65 |
| TRIP12 | 50 | 360 | 1.99 | 4.40E-07 | 0.72 | 0.72 | 0.68 |  | DCBLD2 | 44 | 366 | 2.07 | 5.30E-06 | 0.71 | 0.75 | 0.66 |
| PCYT1A | 40 | 370 | 2.02 | 4.60E-07 | 0.74 | 0.71 | 0.73 |  | SPTB | 49 | 361 | 1.79 | 5.40E-06 | 0.70 | 0.64 | 0.71 |
| PRPF8 | 54 | 356 | 2 | 4.60E-07 | 0.71 | 0.74 | 0.67 |  | EP300 | 51 | 359 | 1.74 | 5.50E-06 | 0.70 | 0.72 | 0.61 |
| BAZ2B | 39 | 371 | 2.1 | 4.80E-07 | 0.74 | 0.72 | 0.72 |  | NBAS | 53 | 357 | 2 | 5.50E-06 | 0.69 | 0.74 | 0.60 |
| ANK3 | 68 | 342 | 2 | 4.90E-07 | 0.69 | 0.74 | 0.63 |  | EIF5B | 40 | 370 | 1.98 | 5.50E-06 | 0.72 | 0.73 | 0.65 |
| FN1 | 69 | 341 | 1.93 | 4.90E-07 | 0.69 | 0.74 | 0.61 |  | ZFC3H1 | 57 | 353 | 1.9 | 5.60E-06 | 0.69 | 0.72 | 0.60 |
| YTHDC1 | 37 | 373 | 2.1 | 4.90E-07 | 0.75 | 0.72 | 0.73 |  | USP42 | 28 | 382 | 1.87 | 5.70E-06 | 0.76 | 0.72 | 0.71 |
| LAMB4 | 39 | 371 | 2.03 | 5.40E-07 | 0.74 | 0.70 | 0.72 |  | PCNT | 60 | 350 | 2.06 | 5.70E-06 | 0.68 | 0.77 | 0.60 |
| MINA | 38 | 372 | 2.04 | 5.60E-07 | 0.75 | 0.72 | 0.74 |  | PTPRCAP | 33 | 377 | 1.93 | 5.80E-06 | 0.74 | 0.64 | 0.82 |
| FBXO38 | 41 | 369 | 2.02 | 5.60E-07 | 0.74 | 0.73 | 0.71 |  | SNORA48 | 46 | 364 | 1.94 | 5.80E-06 | 0.70 | 0.62 | 0.72 |
| NF1 | 77 | 333 | 1.91 | 5.80E-07 | 0.68 | 0.71 | 0.61 |  | RACGAP1 | 27 | 383 | 1.95 | 5.90E-06 | 0.76 | 0.72 | 0.78 |
| FXR1 | 33 | 377 | 2.19 | 5.80E-07 | 0.76 | 0.71 | 0.70 |  | STK38L | 38 | 372 | 2.11 | 5.90E-06 | 0.72 | 0.72 | 0.68 |
| SLC3A1 | 49 | 361 | 1.91 | 6.60E-07 | 0.72 | 0.46 | 0.90 |  | APPL1 | 28 | 382 | 2.01 | 6.00E-06 | 0.76 | 0.72 | 0.75 |
| EP400 | 59 | 351 | 2 | 6.90E-07 | 0.70 | 0.73 | 0.64 |  | CAMSAP3 | 30 | 380 | 1.95 | 6.00E-06 | 0.75 | 0.71 | 0.73 |
| SHPRH | 48 | 362 | 2.02 | 7.70E-07 | 0.72 | 0.72 | 0.67 |  | HIF1A | 38 | 372 | 2 | 6.20E-06 | 0.72 | 0.60 | 0.82 |
| DNAJC18 | 30 | 380 | 2.4 | 7.80E-07 | 0.77 | 0.72 | 0.73 |  | HIF1A-AS2 | 38 | 372 | 2 | 6.20E-06 | 0.72 | 0.60 | 0.82 |
| ARFGEF1 | 60 | 350 | 2.05 | 8.00E-07 | 0.70 | 0.73 | 0.63 |  | DENND1B | 34 | 376 | 2.05 | 6.20E-06 | 0.73 | 0.93 | 0.44 |
| C2ORF42 | 26 | 384 | 2.72 | 8.20E-07 | 0.79 | 0.71 | 0.85 |  | MIS18BP1 | 34 | 376 | 2.6 | 6.30E-06 | 0.73 | 0.81 | 0.59 |
| SDK2 | 31 | 379 | 2.36 | 8.70E-07 | 0.77 | 0.71 | 0.74 |  | GUCY2C | 22 | 388 | 2.54 | 6.30E-06 | 0.79 | 0.70 | 0.77 |
| SRGAP1 | 24 | 386 | 1.96 | 9.30E-07 | 0.80 | 0.66 | 0.92 |  | NOC4L | 30 | 380 | 2.19 | 6.30E-06 | 0.75 | 0.74 | 0.70 |
| EXOC3L1 | 28 | 382 | 2.42 | 9.40E-07 | 0.78 | 0.74 | 0.79 |  | KMT2B | 52 | 358 | 1.85 | 6.30E-06 | 0.69 | 0.72 | 0.60 |
| ATXN2L | 36 | 374 | 1.97 | 1.00E-06 | 0.75 | 0.75 | 0.67 |  | PCDHGB3 | 145 | 265 | 1.63 | 6.40E-06 | 0.63 | 0.77 | 0.48 |
| IFT88 | 28 | 382 | 2.25 | 1.10E-06 | 0.78 | 0.71 | 0.82 |  | BCOR | 31 | 379 | 2.01 | 6.40E-06 | 0.74 | 0.57 | 0.84 |
| SPIDR | 42 | 368 | 2.02 | 1.10E-06 | 0.73 | 0.59 | 0.79 |  | ERI3 | 34 | 376 | 1.87 | 6.50E-06 | 0.73 | 0.72 | 0.74 |
| CAD | 52 | 358 | 1.87 | 1.10E-06 | 0.71 | 0.73 | 0.62 |  | NCOA2 | 43 | 367 | 2.05 | 6.60E-06 | 0.71 | 0.77 | 0.63 |
| HELLS | 32 | 378 | 2.52 | 1.30E-06 | 0.76 | 0.79 | 0.69 |  | MIR6732 | 23 | 387 | 2.26 | 6.70E-06 | 0.78 | 0.67 | 0.87 |
| MAN2B2 | 29 | 381 | 2.64 | 1.30E-06 | 0.77 | 0.71 | 0.76 |  | ZC3H12A | 23 | 387 | 2.26 | 6.70E-06 | 0.78 | 0.67 | 0.87 |
| DNAH10 | 58 | 352 | 1.97 | 1.30E-06 | 0.70 | 0.73 | 0.62 |  | YME1L1 | 27 | 383 | 2.09 | 6.80E-06 | 0.76 | 0.71 | 0.74 |
| NUBP1 | 37 | 373 | 1.98 | 1.40E-06 | 0.74 | 0.72 | 0.73 |  | CNOT1 | 53 | 357 | 1.95 | 7.00E-06 | 0.69 | 0.71 | 0.66 |
| SRCAP | 59 | 351 | 1.82 | 1.40E-06 | 0.70 | 0.73 | 0.61 |  | RPGRIP1L | 29 | 381 | 2.03 | 7.00E-06 | 0.75 | 0.71 | 0.79 |
| EEA1 | 44 | 366 | 2.1 | 1.50E-06 | 0.72 | 0.73 | 0.68 |  | DDX39A | 31 | 379 | 1.89 | 7.10E-06 | 0.74 | 0.49 | 0.90 |
| GEMIN5 | 33 | 377 | 2.29 | 1.50E-06 | 0.75 | 0.73 | 0.79 |  | ENPP3 | 46 | 364 | 2.24 | 7.40E-06 | 0.70 | 0.75 | 0.61 |
| NUP188 | 60 | 350 | 1.97 | 1.50E-06 | 0.69 | 0.58 | 0.73 |  | CORO1B | 31 | 379 | 1.95 | 7.50E-06 | 0.74 | 0.70 | 0.77 |
| RAD54L | 32 | 378 | 2.08 | 1.50E-06 | 0.76 | 0.59 | 0.84 |  | EDRF1 | 40 | 370 | 1.96 | 7.50E-06 | 0.72 | 0.70 | 0.70 |
| SPEN | 66 | 344 | 1.86 | 1.50E-06 | 0.69 | 0.72 | 0.62 |  | PCDHGA5 | 152 | 258 | 1.62 | 7.50E-06 | 0.63 | 0.75 | 0.49 |
| KDM6B | 33 | 377 | 2.19 | 1.60E-06 | 0.75 | 0.80 | 0.67 |  | TRPM6 | 47 | 363 | 1.76 | 7.60E-06 | 0.70 | 0.43 | 0.94 |
| MED12L | 91 | 319 | 1.91 | 1.80E-06 | 0.66 | 0.74 | 0.57 |  | EYA2 | 28 | 382 | 1.86 | 7.60E-06 | 0.75 | 0.66 | 0.79 |
| IQGAP1 | 34 | 376 | 2.46 | 1.80E-06 | 0.75 | 0.78 | 0.71 |  | P2RY12 | 37 | 373 | 1.97 | 7.70E-06 | 0.72 | 0.66 | 0.76 |
| ITGA7 | 33 | 377 | 2.13 | 1.80E-06 | 0.75 | 0.60 | 0.85 |  | ARHGEF11 | 32 | 378 | 2.47 | 7.80E-06 | 0.74 | 0.76 | 0.72 |
| TM9SF2 | 35 | 375 | 1.98 | 1.90E-06 | 0.74 | 0.73 | 0.74 |  | CCDC88A | 37 | 373 | 2.1 | 7.80E-06 | 0.72 | 0.72 | 0.68 |
| STAB1 | 64 | 346 | 1.9 | 2.00E-06 | 0.69 | 0.73 | 0.59 |  | XRN1 | 47 | 363 | 1.89 | 7.80E-06 | 0.70 | 0.70 | 0.66 |
| LRRC41 | 27 | 383 | 1.97 | 2.10E-06 | 0.77 | 0.48 | 0.96 |  | CEP350 | 36 | 374 | 1.94 | 8.00E-06 | 0.73 | 0.51 | 0.92 |
| GPR107 | 28 | 382 | 2.71 | 2.20E-06 | 0.77 | 0.74 | 0.75 |  | NAPEPLD | 30 | 380 | 1.89 | 8.30E-06 | 0.74 | 0.71 | 0.73 |
| SNORA15 | 30 | 380 | 1.97 | 2.20E-06 | 0.76 | 0.74 | 0.73 |  | CP | 32 | 378 | 2.19 | 8.40E-06 | 0.74 | 0.69 | 0.72 |
| DMD | 95 | 315 | 1.79 | 2.20E-06 | 0.66 | 0.63 | 0.62 |  | PCDHGA9 | 109 | 301 | 1.82 | 8.40E-06 | 0.64 | 0.75 | 0.53 |
| AVEN | 27 | 383 | 2.45 | 2.30E-06 | 0.77 | 0.72 | 0.74 |  | JAK2 | 27 | 383 | 1.98 | 8.60E-06 | 0.76 | 0.67 | 0.81 |
| PRPF40B | 33 | 377 | 2.16 | 2.30E-06 | 0.75 | 0.70 | 0.76 |  | AP1G1 | 41 | 369 | 1.94 | 8.70E-06 | 0.71 | 0.73 | 0.66 |
| MON2 | 30 | 380 | 2.26 | 2.40E-06 | 0.76 | 0.73 | 0.77 |  | FASTKD2 | 32 | 378 | 2.42 | 8.70E-06 | 0.74 | 0.81 | 0.69 |
| DOCK3 | 64 | 346 | 1.98 | 2.50E-06 | 0.69 | 0.74 | 0.66 |  | TVP23A | 28 | 382 | 2.35 | 8.80E-06 | 0.75 | 0.71 | 0.79 |
| ARID1A | 95 | 315 | 1.79 | 2.60E-06 | 0.66 | 0.77 | 0.48 |  | PCDHGA2 | 174 | 236 | 1.52 | 8.90E-06 | 0.63 | 0.75 | 0.47 |
| PLS3 | 22 | 388 | 2.55 | 2.60E-06 | 0.80 | 0.71 | 0.86 |  | PCDHGA3 | 174 | 236 | 1.52 | 8.90E-06 | 0.63 | 0.75 | 0.47 |
| TATDN2 | 34 | 376 | 2.31 | 2.60E-06 | 0.74 | 0.73 | 0.74 |  | NPAT | 40 | 370 | 1.83 | 9.00E-06 | 0.71 | 0.71 | 0.65 |
| DMXL2 | 52 | 358 | 2.06 | 2.60E-06 | 0.70 | 0.73 | 0.63 |  | BRAF | 45 | 365 | 1.93 | 9.30E-06 | 0.70 | 0.73 | 0.69 |
| PARP10 | 26 | 384 | 2.23 | 2.60E-06 | 0.78 | 0.75 | 0.73 |  | KIAA0556 | 41 | 369 | 1.97 | 9.30E-06 | 0.71 | 0.59 | 0.83 |
| CCDC18 | 45 | 365 | 2.03 | 2.70E-06 | 0.71 | 0.71 | 0.69 |  | PLEKHA5 | 41 | 369 | 2.1 | 9.70E-06 | 0.71 | 0.77 | 0.61 |
| ADAMTS18 | 58 | 352 | 2.04 | 2.70E-06 | 0.69 | 0.51 | 0.84 |  | JMJD7-PLA2G4B | 28 | 382 | 2.27 | 9.70E-06 | 0.75 | 0.80 | 0.61 |
