# Supplementary material for: Mutations Defining Patient Cohorts With Elevated PD-L1 Expression in Gastric Cancer
Source: Front Pharmacol. 2019 Jan 8;9:1522. doi: 10.3389/fphar.2018.01522 (PMC6331584; doi:10.3389/fphar.2018.01522)
Supplement: Supplementary file 3 [file Table_3.DOCX]

**Supplemental Table 3. Gene ontology analysis** of the mutant genes significantly associated with increased PD-L1 (*CD274*) expression after step-up multiple testing correction.

| **Function** | ***p*-value** | **Genes included** |
| --- | --- | --- |
| regulation of gene expression | 0.00015 | DDX39A, HIF1A, MCPH1, NF1, NPAT, PIK3CA, ZC3H12A, SART3 |
| microtubule-based movement | 0.00034 | DNAH10, KIF5B, KIF15, DNAH2, KIF9, RACGAP1, DNAH8 |
| homophilic cell adhesion via plasma membrane adhesion molecules | 0.00049 | SDK2, PCDHGA9, PCDHGA7, PCDHGA6, PCDHGA5, PCDHGB3, PCDHGA3, PCDHGA2, PCDHGB4 |
| cellular response to DNA damage stimulus | 0.00069 | DDX39A, SHPRH, FBXO18, UBR5, ZC3H12A, SMC6, SPIDR, POLQ, ATM, TRIP12 |
| double-strand break repair via homologous recombination | 0.00161 | FBXO18, SMC6, SPIDR, POLQ, RAD54L, ATM |
| positive regulation of GTPase activity | 0.00228 | RAB3GAP1, NF1, DOCK8, DENND2A, RACGAP1, HERC1, ARFGEF1, DOCK3, IQGAP1, ARHGEF11, DENND1B, P2RY12, TBCD, JAK2, SRGAP1, SPTB |
| chromatin-mediated maintenance of transcription | 0.00449 | KMT2B, ARID1A, ARID1B |
| DNA-repair | 0.00589 | EYA2, SHPRH, UBR5, TRRAP, POLQ, RAD54L, FANCA, ATM, TRIP12 |
